# Clinical, immunological, and virological outcomes of pediatric antiretroviral therapy in central China

**DOI:** 10.1186/1756-0500-7-419

**Published:** 2014-07-03

**Authors:** Junwen Zheng, Dongchi Zhao

**Affiliations:** 1 Department of Pediatrics, Zhongnan Hospital of Wuhan University, Wuhan 430071, China

**Keywords:** HIV/AIDS, Medical practice, Pediatrics, Antiretroviral therapy, Infectious diseases outcomes

## Abstract

**Background:**

Antiretroviral therapy (ART) reduces HIV-related mortality and morbidity substantially in children. The clinical characteristics, immunological and virological outcomes were evaluated in HIV-infected children receiving ART.

**Methods:**

Twenty-six HIV-1-infected children receiving ART in Hubei province, China, were enrolled retrospectively in this study. During the period of ART, plasma viral load, lymphocyte phenotype of CD4 and CD8 cells and clinical events were assessed.

**Results:**

The median duration of ART was 41 months (18–72.3 months). In children showing clinical improvement, high viral suppression rate below log10 (2.7) copies/ml by the third months of ART was observed. The median CD4 cell counts reached to 820.5/μl by 12 months and the median ratio of CD4/CD8 increased to 0.6 by 21 months. The counts of peripheral white blood cells and red blood cells decreased in the first 12 months, while Hb concentration, MCV and MCH increased (*P* < 0.001).

**Conclusions:**

Despite the limited small sample size, ART is an effective strategy for inhibiting HIV replication and reconstructing the immunological response in children with AIDS.

## Background

Antiretroviral therapy (ART) reduces HIV-related mortality and morbidity substantially in children, and without HIV care, the progression of HIV-infection children is particularly aggressive. The World Health Organization (WHO) guidelines recommended initiating ART in all children according to their clinical stage and CD4 cell count or percentage [1]. However, long-term therapy can adversely affect a child’s quality of life because of the regimen’s side effects [2] and ART resistance [3] which may result in growth faltering, stunting, especially once they reach adolescence [4,5].

The Division of Treatment and Care (DTC) of the National Center for AIDS/STD Control of China estimated nearly 6000 children infected with HIV-1 in China at the end of 2011 [6]. Before 2005, few HIV-infected children in China received ART, and pediatric formulations were unavailable by referring to adult fixed-dose combinations [7-9]. The DTC initiated the National Pediatric Free ART Program (NPFAP) in June 2005. Hubei, a central province in China, was one of the first eight trial units to undertake this program. In the present study the short term virological, immunological and clinical outcomes of HIV infected children with ART was evaluated from pediatric HIV care program in central China.

## Methods

Enrolment and study setting based on the WHO clinical and laboratory criteria [1], 26 of 68 HIV-1-infected Chinese children were eligible for free ART formulations and enrolled in this cohort. The NPFAP was performed at the HIV/AIDS Treatment Center of Zhongnan Hospital, Wuhan University in Hubei province, China. This programme is supported by the Chinese Center of Disease Control, which started to provide free access to pediatric antiretroviral therapy in China. The programme included prophylaxis and treatment for opportunistic infections, psychosocial support, nutritional support, and antiretroviral treatment.

ART eligibility was defined based on the WHO guidelines [1]. HIV-infected children presenting with WHO stage I or II were eligible for ART if the CD4 cell counts <350 cells/μl or CD4% <15% for children >3 years old, CD4 cell counts <750 cells/μL or CD4% <20% for children 1–3 years old, or virologically diagnosed children <1 year old regardless of CD4 cell count or CD4%. Children presenting with WHO stage III/IV were eligible irrespective of their CD4 percentage or CD4 count. First-line ART comprised the nucleoside reverse transcriptase inhibitors (NRTIs) zidovudine (AZT) or stavudine (D4T) plus lamivudine (3TC), and the non-nucleoside reverse transcriptase inhibitors (NNRTIs) nevirapine (NVP) or efavirenz (EFV) for all eligible children. The protease inhibitor (PI) lopinavir/ritonavir was as the second line preparation. Of 26 HIV-infected children, eight had been treated with D4T, 3TC and NVP or EFV, and their treatments were adjusted to the free regimens of AZT, 3TC and NVP at the time of entry into the programme of eighteen ART-naïve HIV children in 2005. The clinical and laboratory data collected at baseline were analyzed for all children enrolled.

### Laboratory data collection

Blood samples were collected in the morning and processed the same day at Wuhan University HIV/AIDS Treatment Center. CD4 and CD8 cell counts were measured using the single-platform assay in a FACSCalibur flow cytometer (Becton Dickinson, Aalst, Belgium). The CD4 percentage was calculated using the formula: CD4% = absolute CD4 count/(white blood cell (WBC) count × percentage of lymphocytes). An absorbance cytochemistry and haematology analyser (Coulter Beckman, Fullerton, CA, USA) was used to measure haematological parameters including hemoglobin concentration (Hb), red blood cell (RBC), and red cell indices such as mean corpuscular volume (MCV), mean corpuscular Hb content (MCH), as well as mean corpuscular Hb concentration (MCHC). Viral load (VL) was detected by a real-time reverse transcriptase polymerase chain reaction (Roche Molecular Systems, Branchburg, NJ, USA). Therapeutic failure was defined as a low CD4 cell count according to the WHO criteria or an increase in VL to > log10 (3.7) copies/ml [1]. The limit of quantification (LOQ) was log10 (2.7) copies/ml. Body weight and height were collected and monitored at baseline, monthly in first three months, and every three months thereafter.

This program used the Free ART database to monitor current and past patients in this Program. Information was collected at the time of treatment initiation, each follow-up visit, regimen change, and treatment termination.

### Data analysis

Analysis of data was performed by on-treatment analysis of the observed data. Z-scores (standard deviation scores) are calculated as (variable-mean)/standard deviation. Statistical analysis was performed using SPSS 19.0 software (SPSS Inc, Chicago, IL, USA). All tests were two-tailed with *P-values* <0.05 considered significantly.

### Ethical approval

This study was approved by the Institutional Review Board of the National Center for AIDS/STD Control and Prevention, Chinese Center for Disease Control and Prevention. All parents of patients signed informed consent for participation.

## Results

### Characteristics of HIV-1-infected children

General characteristics are shown in Table 1. Eight (31%) HIV-1-infected children received ART (D4T, 3TC and EFV) at baseline for 1–3 years until June 2005, and then their therapy was changed into the free ART regimens of AZT, 3TC and NVP. Six children had been diagnosed with AIDS stage III–IV and the other twenty children had moderate-to-severe immunosuppression and a CD4 T cell percentage <15-20%. Twenty-four (92%) of the 26 children had a detectable VL > log10 (3.7) copies/ml. The median age at baseline was 7.7 years (1.7–12 years), and the latent infection time was 6.8 years (1.4–11.5 years). Nine children had been infected through blood products or transfusion, and four of these had coinfected hepatitis C virus. Seventeen children had been infected through mother-to-child transmission. The median duration of administration of ART was 41 months. In the follow up time, two (8%) children died (one due to severe diarrhea diseases and by non-AIDS related cases). One child had an immunological and virological failure and the second line ART regime contains NVP plus lopinavir/ritonavir was initiated after 41 months on ART. By the end of a median time of 41 months, 24 children (92%) were alive and receiving ART.

**Table 1:** **Characteristics of children started on antiretroviral treatment (*n*** **= 26)**

**Characteristics**	**Numbers(interval)**	**Percentage**
Age at start ART (years, median)^a^	7.7 (1.5–12)	
<2 years	2	7.7%
2–4.9 years	4	15.3%
5–14 years	20	77%
Sex (male/female)	16/10	61.5%/38.5%
Reason for ART		
WHO stage III-IV	6	36.5%
Baseline CD4 count <15%	20	63.5%
Naïve ART regimen		
D4T/3TC/NVP (EFV)	8	30.8%
AZT/3TC/NVP	18	69.2%
Time on ART (months, median)^a,b^	41 (18–72.3)	
<2 years^b^	5	19.2%
2 years to <3 years^b^	7	26.9%
3 years to <4 years^b^	6	23.1%
>4 years^b^	8	30.8%
HIV transmission		
Blood product or transfusion recipient	9	34.6%
Mother-to-child transmission	17	65.4%

^a^Values are expressed as median (interquartile range).

^b^As of 30 December 2008 (closing date of dataset).

WHO, World Health Organization; ART, antiretroviral therapy; D4T, stavudine; 3TC, lamivudine; AZT, zidovudine;NVP, nevirapine; EFV, efavirenz.

### ART and immunologic outcomes

**Figure 1:**
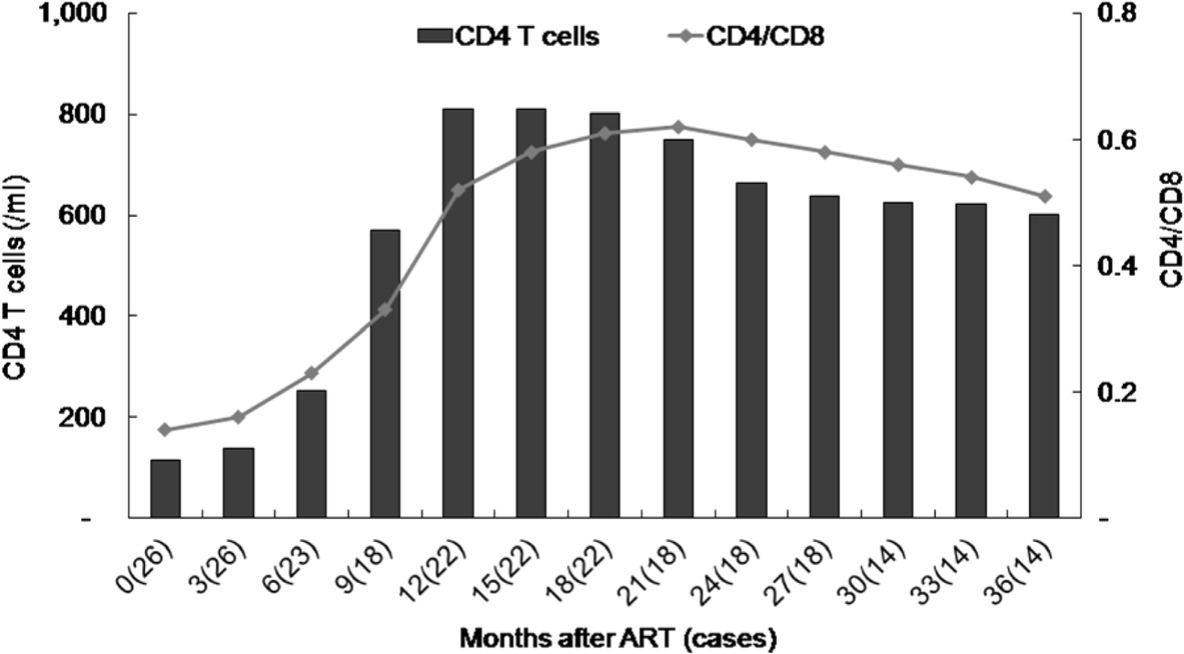
**Antiretroviral therapy and CD4**^**+ **^**T cell counts.** Data exclude two children who had ART treatment failure and shown as median.

The baseline median CD4 count was 115.7 cells/μl, and this count increased markedly to 268.5 cells/μl in the third month and reached its highest level of 810.4 cells/μl at the first year of ART (Figure 1). The CD4 count increased to 1.8-fold by the middle of the first month and reached its highest level of 6.9-fold in the ninth month. CD4 percentage increased from 12% to 25% in the sixth month and was maintained >34% in the ninth month. Two patients demonstrated failure to ART in the third year. Before the free ART program, both had been treated with D4T, 3TC and NVP/EFV, and their CD4 cell counts decreased to 95 and 97 cells/μl at the end of the third year when they showed resistance to the AZT/3TC/NVP protocol. One died of profound diarrhea after 42 months of ART. The other was given NVP plus LPV/RTV, and the absolute CD4 cell count increased markedly from 10 to 750 cells/μl within six months. Twenty-four (92%) of the 26 HIV-infected children achieved immunological advantage from this free ART.

### ART and virological outcomes

**Figure 2:**
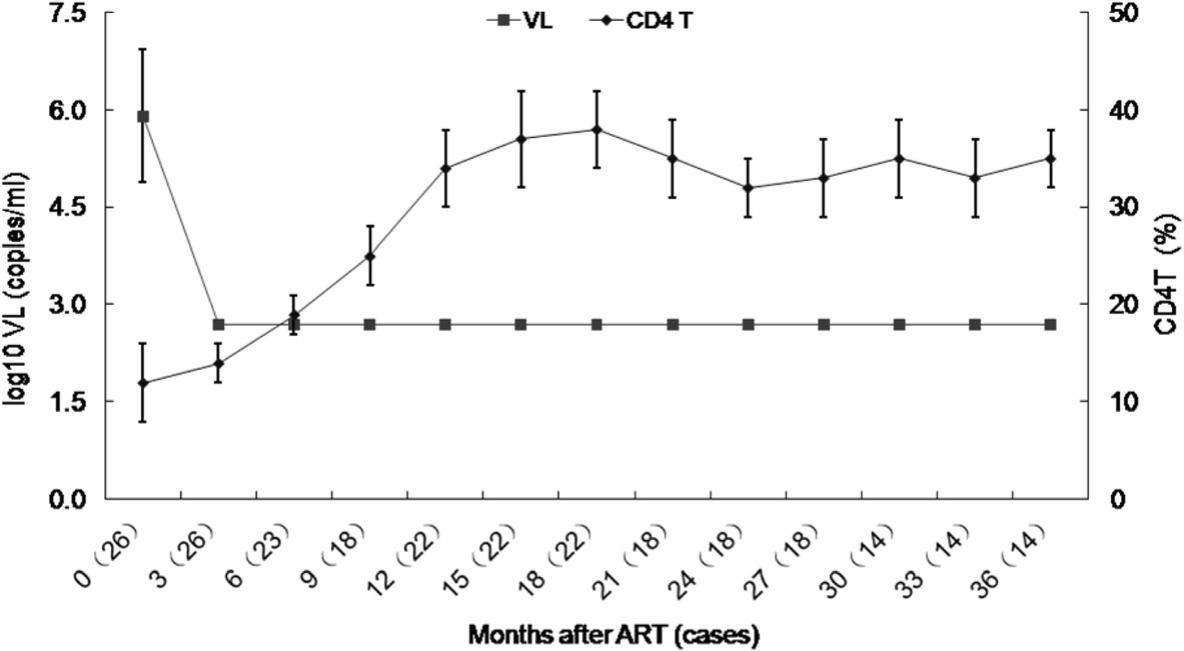
**Summary of viral load and the percentage of CD4 T cell during follow-up after antiretroviral therapy.** The values are the mean of log 10 (copies/ml) and CD4 T cell percentage.

At the beginning of this study, the VL mean was log 10 (5.9 ± 3.2) copies/ml. In 23 children, the VL decreased to less than log10 (2.7) copies/ml within 3 months of ART and VL was maintained at this level until the preparation of this study (Figure 2). Two HIV-infected children who had received ART before the free regimen appeared to treatment failure with high VL levels > log10 (4.7) copies/ml at 36 months. One was given NVP plus LPV/RTV, and the VL decreased to an undetectable level after three months, while the CD4 count increased to 750/μl after six months. As results, the combination of AZT/3TC/NVP was effective in suppressing HIV replication in most HIV infected children.

### ART and growth

**Figure 3:**
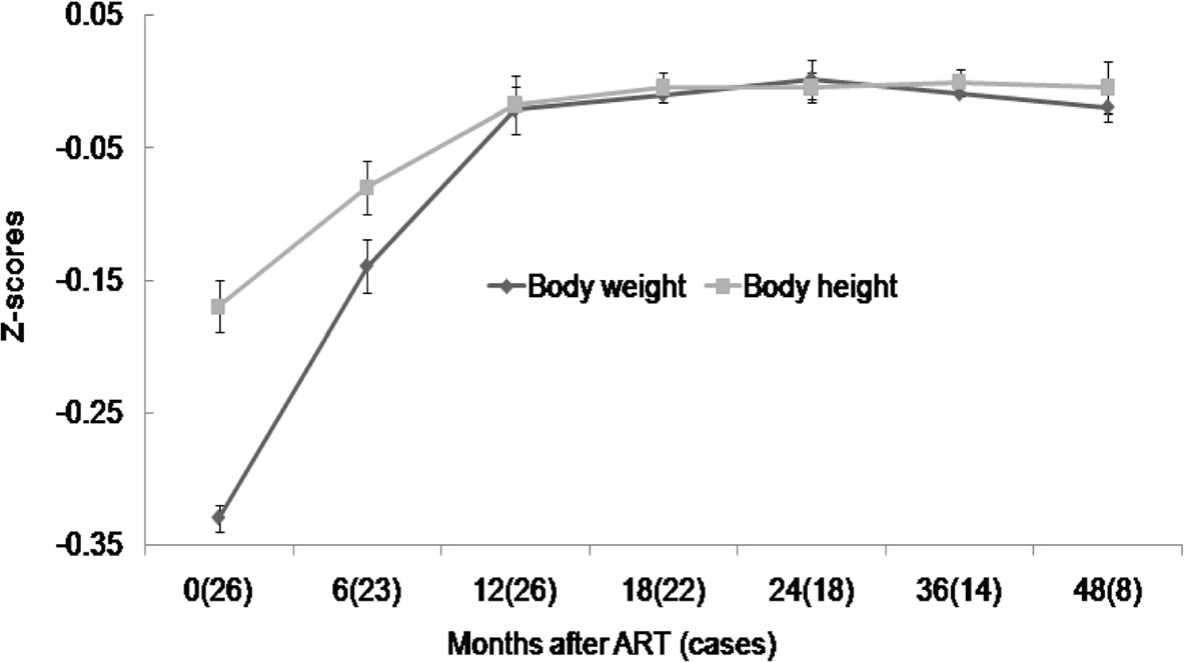
Changes of mean Z-scores for body weight and height for age in children receiving antiretroviral therapy.

At the beginning of ART, the mean Z-scores of HIV-1-infected children’s body weight and height for age were –0.33 and –0.17 (Figure 3), compared those with the general population standards of Chinese children (P < 0.001). Figure 3 shows that 23 of the 26 children achieved quick recovery growth in the first year, after which their growth velocity was the same as in healthy childhood. The two children virological failure with increased VL stunted growth had Z-scores of –0.35, –0.33 and –0.26, –0.23 for weight and height after the third year of ART. One was changed to NVP plus LPV/RTV and recovered a normal growth rate. By the end of 41 months of time, the increase in body weight and height of 23 children was associated with positive clinical outcomes.

### ART and adverse events

**Figure 4:**
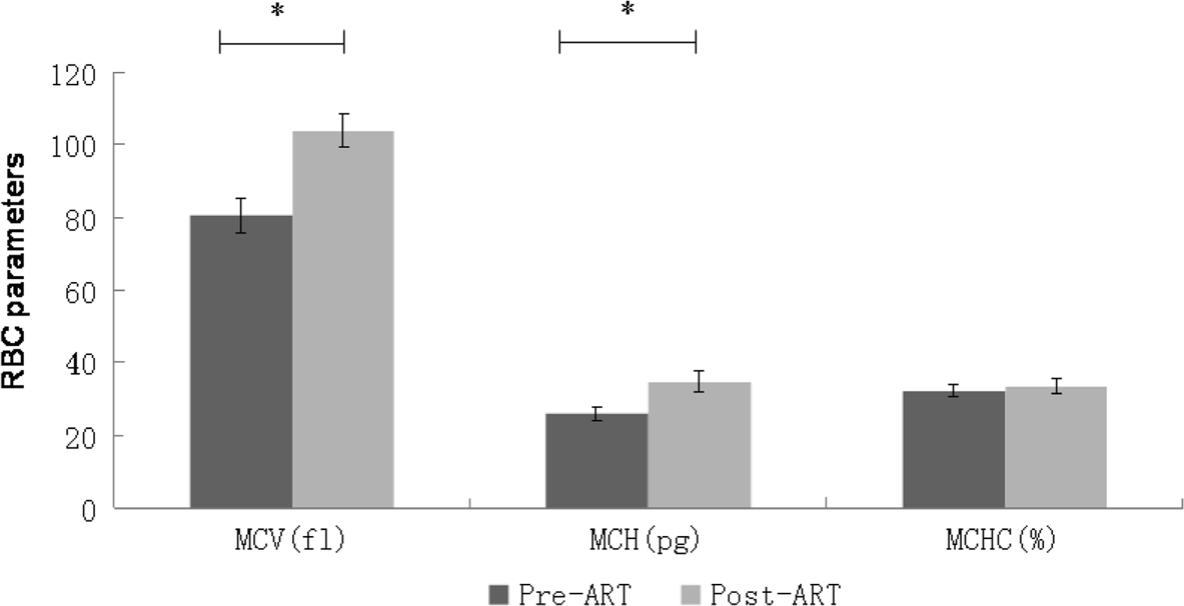
**Changes in red blood cell parameters in children receiving antiretroviral therapy.** RBC, red blood cell; MCV, mean corpuscular volume; MCH, mean corpuscular Hb content, and MCHC, mean corpuscular Hb concentration. The values are expressed as mean ± SD. * P < 0.001.

All children had mild to moderate anemia at the beginning of ART. At the end of the first year of ART, Mean Hb had increased from 107.9 ± 13.7 g/L to 112.2 ± 11.2 g/L (P < 0.001). In contrast, Mean of red blood cell count decreased from 4.0 ± 0.4 × 10^12^/L to 3.5 ± 0.4 × 10^12^/L (P < 0.001). All children demonstrated a change to megaloblast-like red blood cell (Figure 4). The baseline means of WBC and the lymphocyte counts were 5.1 ± 1.3 × 10^9^/L and 2.0 ± 0.7 × 10^9^/L. After 12 months of ART, the WBC count decreased to 4.4 ± 0. 8 × 10^9^/L (P = 0.04), and the lymphocyte count decreased to 1.8 ± 0.6 × 10^9^/L (P = 0.02). Of the patients with severe anemia, none required a change of regimen because of symptomatic anemia or blood transfusions. Other haematological parameters evaluation included macrocytosis and thrombocytopenia (Figure 4). Macrocytosis was identified in all patients on ART compared with their baseline level (P < 0.001). All patients had an MCV >100 fl. However, there was no clinical consequence of macrocytosis in these patients. No patient exhibited thrombocytopenia or liver dysfunction during ART.

## Discussion

Our study shows that ART (AZT/3TC/NVP) should be effective in achieving an adequate response in HIV-infected children. Most HIV-children showed improved clinical, immunological and virological outcomes. Such therapy results are similar to those reported for adults [10] and a small cohort of older children in resource-limited settings [11]. These responses were greater than those reported in severe immunosuppression settings [12,13].

The immunological outcomes from receiving ART showed evidence of significant immunological reconstitution in most of the cohort. After 12 months of ART, most HIV-infected children with available CD4 cell percentage data attained near-normal immune status (e.g. CD4 cell percentage of 34%). The CD4 cell counts increased to the highest level in the first year, and children with the most profound immunosuppression at the onset had the greatest increases in CD4 cell count (from 17 to 510 cells/μl). However, these children still had the lowest absolute CD4 cell counts, and a significantly higher proportion experienced an opportunistic infection. This suggests that, although immune function improves significantly in these children irrespective of the baseline CD4 cell count and percentage, the greater the baseline immunodeficiency, the more likely the patient will remain at risk of opportunistic infections during the first year of receiving ART [14].

The survival rate is determined mainly by age-risk in the first six months of ART, whereas the patient’s sex, baseline CD4 cell percentage, baseline WHO clinical stage and initial drug regimens are not significantly associated with death in different age groups [15]. Our data show a survival probability of 92.3%, which is greater than that reported in large trials in Africa [16]. One reason for this difference could be the small samples size in our study and that most of these HIV-infected children were older than two years.

Our results are similar to those from resource-rich settings [17], although the children from resource-rich settings were mainly ART non-native and less immunosuppressed, and were treated mainly with protease inhibitor-containing regimens [2]. These data suggest that better outcomes are achieved regardless of when ART is started provided it is started before profound immunosuppression develops [16]. In resource-limited settings, under routine programme conditions, giving NNRTI-containing ART to severely and profoundly immunosuppressed children can achieve good early outcomes. This provides further impetus to the urgency of ensuring that children in resource-limited settings have early access to life-prolonging ART. Virological evidence is an advantage in assessing therapeutic responses [18]. In this study, the mean baseline VL decreased to below the LOQ after three months of ART. Two children developed virological failure, and their VLs increased after three years of therapy. One child’s regimen was changed by substituting D4T with AZT, but viral replication could not be suppressed and the absolute CD4 cell count declined to 20/μl, and the child died of severe diarrhea in the fourth year. Another child’s regimen was changed to NVP plus lopinavir/ritonavir, to our surprise, the CD4 cell count rebounded from 10/μl to 750/μl, and VL was below the LOQ after six months despite frequent respiratory tract infections. This is important because it suggests that LPV/RTV is more effective in a child undergoing ART with virological and immunological failure [19].

HIV-infected children are significantly shorter and lighter than healthy children, and the growth differences increase with age [4]. Our data show that baseline age Z-scores for weight and height were –0.33 and –0.17 respectively, and that height and weight increased in all children who received ART. Z-scores for weight and height increased to nearly 0 during the three years after initiation of combination therapy. Growth velocity was greater in HIV-infected children with ART than in healthy children especially in the first year of treatment. Initiation of ART improves the mean standardized weight-for-age Z-score and weight-for-age percentiles [20]. These results suggest that combination therapy in severely HIV-infected children helps increase weight and, to a lesser extent, height and that children experience a continued catch-up in weight and height after starting ART [5].

ART causing macrocytic anemia has been reported elsewhere [21]. Macrocytosis was associated with ART, and all children on ART had increase level of MCV and MCH, whereas MCHC did not change. Anemia is common in HIV-infected children, and this probably reflects the multifactorial causes involved in the pathogenesis. ART drugs such as AZT and D4T are associated with a transient macrocytic anemia by inhibiting β-globin gene expression in human erythroid progenitor cells [22,23]. Usually, the hemoglobin A2 concentration may be high enough to lead to a misdiagnosis of β-thalassemia trait if the clinician is not aware of this unexpected effect of HIV infection and its treatment. Despite this, macrocytosis had no clinical consequence and ART increased the CD4 cell count and decreased the total lymphocyte count.

## Conclusions

In conclusion, despite the limited small sample size, ART is an effective strategy for inhibiting HIV replication and reconstructing the immunological response in children with AIDS.

## Abbreviations

ART: Antiretroviral Therapy; NRTIs: Nucleoside Reverse Transcriptase Inhibitors; NNRTIs: Non-nucleoside Reverse Transcriptase Inhibitors; VL: Viral Load.

## Competing interests

The authors declare that they have no competing interests.

## Authors’ contributions

ZJ and ZD wrote the manuscript and coordinated the NPFAP project, collected the data. Both authors read and approved the final manuscript.
